# Antioxidant and Antiproliferative Activities of Leaf Extracts from *Plukenetia volubilis* Linneo (Euphorbiaceae)

**DOI:** 10.1155/2013/950272

**Published:** 2013-09-18

**Authors:** Ana Karina Lima Nascimento, Raniere Fagundes Melo-Silveira, Nednaldo Dantas-Santos, Júlia Morais Fernandes, Silvana Maria Zucolotto, Hugo Alexandre Oliveira Rocha, Katia Castanho Scortecci

**Affiliations:** ^1^Departamento de Biologia Celular e Genética, Centro de Biociências, Universidade Federal do Rio Grande do Norte, 59072-970 Natal, RN, Brazil; ^2^Laboratório de Biotecnologia de Polímeros Naturais-Biopol, Departamento de Bioquímica, Centro de Biociências, Universidade Federal do Rio Grande do Norte, 59072-970 Natal, Brazil; ^3^Departamento de Farmácia, Centro de Ciências da Saúde, Universidade Federal do Rio Grande do Norte, 59010-180 Natal, Brazil

## Abstract

*Plukenetia volubilis* Linneo, or Sacha inca, is an oleaginous plant from the Euphorbiaceae family. The aim of this work was to perform a chemical and biological analysis of different leaf extracts from *P. volubilis* such as aqueous extract (AEL), methanol (MEL), ethanol (EEL), chloroform (CEL), and hexane (HEL). Thin layer chromatography analysis revealed the presence of phenolic compounds, steroids, and/or terpenoídes. Furthermore, the antioxidant activities were analyzed by *in vitro* assays and their effects on cell lineages by *in vivo* assays. The Total Antioxidant Capacity (TCA) was expressed as equivalent ascorbic acid (EEA/g) and it was observed that the extracts showed values ranging from 59.31 to 97.76 EAA/g. Furthermore, the DPPH assay values ranged from 62.8% to 88.3%. The cell viability assay showed that the extracts were able to reduce viability from cancer cells such as HeLa and A549 cells. The extracts MEL and HEL (250 *µ*g/mL) were able to reduce the proliferation of HeLa cells up to 54.3% and 48.5%, respectively. The flow cytometer results showed that these extracts induce cell death via the apoptosis pathway. On the other hand, the extracts HEL and AEL were able to induce cell proliferation of normal fibroblast 3T3 cells.

## 1. Introduction

Natural products have long been investigated for their potential benefits. In the 1900s most medicines were obtained from the cooking, infusion, or maceration of roots, barks, leaves, or flowers. Today, natural products still have a huge importance as a source of new drugs and leads. Approximately 60% of anticancer compounds and 75% of drugs for infectious disease come from natural products or their derivatives [1–4]. In order to develop new drugs, different plants are being selected randomly or based on ethnobotanic and ethnopharmacologic knowledge. The extracts obtained from these plants are then evaluated using different *in vitro* assays as well as their effects on normal or tumor cells. Then, the potential extracts are purified in order to obtain their potential fractions or molecules to be tested by *in vitro* and *in vivo* assays again, followed by analysis of their potential for new drugs [5].

Recently, many diseases such as cancer, diabetes, arteriosclerosis, inflammatory disease, autoimmunity, cardiovascular disease, and Alzheimer's have been associated with the increase of reactive oxygen species (ROS) or the inability of the organism to reduce these ROS that were normally produced by the organism cells, a process known as oxidative stress [6]. Antioxidants are important substances that have the ability to protect the organism from the damage caused by the oxidative stress. Due to this ability, there is a special interest in the presence of natural antioxidants in medicinal plants that may help an organism to keep the normal balance of ROS. In the case of cancer, these antioxidant substances are being used in the treatment to prevent the normal cell from becoming a cancer cell as well as to kill cancer cells; furthermore, these compounds had no negative effects on normal cells [7, 8].

The Euphorbiaceae is formed by more than 6,000 species with extreme diversity of secondary compounds produced [9, 10]. This variability of compounds may explain the different uses of the plants from this family such as antiproliferative, antimicrobial, cytotoxic activity, and anti-inflammatory, among others [9]. Moreover, many times, the latex produced by these plants has been used as medicine for skin disease as well as skin healing; for example, *Euphorbia thymifolia, E. neriifolia, and E. nivulia*, as well as the *Croton bonplandianum*, have been used to treat scabies and other skin diseases [11]. 

*Plukenetia volubilis *Linneo or sacha inchi, the target of this work, is a climbing shrub plant from the Euphorbiaceae that grows mostly in the Amazon region [12]. Some studies have shown that seeds from this plant are an excellent source of protein (27–33%) and oil (35–60%) [13]. The oil composition is mostly omega 3 and omega 6 (48.61% and 36.8%, resp.) [13, 14]. Some studies have been done using seeds and leaves in order to reduce the lipid profile from patients with postprandial lipemia [15] and hypercholesterolemia [16]. However, there is no scientific data about the use of leaves for skin disease, as it has been used in folk medicine in the Amazon region. The aim of the work presented here was to evaluate the effects from five extracts obtained from fresh leaves: methanol (MEL), ethanol (EEL), chloroform CEL, hexane (HEL), and aqueous (AEL). These extracts were analyzed using antioxidant assays and antiproliferative assays using normal and tumor cells. The results showed that some of these extracts had antioxidant and antiproliferative activities against HeLa cells. Furthermore, it was observed that the extracts were able to stimulate the cell proliferation in fibroblast cells-3T3.

## 2. Materials and Methods

### 2.1. Plant Material

*Plukenetia volubilis* plants were grown in soil (soil and sand 1 : 1) in Parnamirim, RN, Brazil (−05° 47′ 42′′ +35° 12′ 34′′). After six months, leaves were collected to obtain the different extracts. Two specimens were deposited in a herbarium at Centro de Biociências da Universidade Federal do Rio Grande do Norte (UFRN) under the number 10854. 

### 2.2. Preparation of Leaf Extracts

Fresh leaves were collected, and 15 g was divided into small pieces by hand and then transferred to a flask in a proportion of 1 : 10 (drug : solvent, w/v), using the following solvents: methanol, ethanol, chloroform, hexane, and water. The material was protected from light with aluminum foil, and these flasks were shaken at 50 rpm for 24 h. Then the extracts were filtered on Whatman paper n° 1 and dried in Rotavapor at 40°C, resulting in five extracts: methanol, ethanol, chloroform, hexane, and aqueous. After that, the extracts were weighed and then resuspended into DMSO (CRQ/nuclear) to final concentration of 10 mg/mL (extract stock solution). 

### 2.3. Phytochemistry Screening by Thin Layer Chromatography (TLC)

This assay was conducted by TLC, using aluminum sheets of silica gel F_254_ (Merck). All chromatograms were developed in a saturated chamber. Two systems were used as mobile phases: (1) ethyl acetate : formic acid : water : methanol (10 : 0.5 : 0.6 : 0.2) and (2) toluene : ethyl acetate : methanol (5 : 5 : 0.5). The eluent systems employed in this study were selected or developed according to data from the literature [17]. After the chromatograms were developed, the plates were dried and the spots were visualized sequentially under UV light at 254 and 365 nm. The plates were then sprayed with specific chromogenic agents according to the chemical substances analyzed. The following solutions were used as visualizing agents: vanillin sulfuric +105°C, natural reagent A 0.5%, ferric chloride 1% in methanol, and Dragendorff reagent. Kaempferol, luteolin, and apigenin, all obtained from Sigma-Aldrich, were used as standard sample.

### 2.4. Total Content of Sugar, Protein, and Phenolic Compounds

Total sugar was estimated by the phenol-H_2_SO_4_ reaction using D-glucose (Sigma-Aldrich) as a standard [18]. Protein content was measured using Spector's method with bovine albumin as a standard [19]. Total phenolic compounds in the extract solution were determined using Folin-Ciocalteu's coloimetric method and gallic acid as a standard [20]. 

### 2.5. Antioxidant Activity

The antioxidant activity was examined by conducting *in vitro* tests: total antioxidant capacity, hydroxyl radical scavenging, superoxide radical scavenging, ferric chelating, and reducing power, as previously described in [21, 22].

#### 2.5.1. Determination of Total Antioxidant Capacity (TAC)

The assay for total antioxidant capacity is based on the reduction of Mo^+6^ to Mo^+5^ by the plant extracts and subsequent formation of green phosphate/Mo^+5^ complexes at acid pH [23]. The tubes containing the plant extracts at 250 *μ*g/mL concentration and the reagent solution (0.6 M sulfuric acid, 28 mM sodium phosphate, and 4 mM ammonium molybdate) were incubated at 95°C for 90 min. After this time, the mixture was cooled at room temperature and the absorbance of each tube was measured at 695 nm against a blank. The antioxidant capacity was expressed as mg of ascorbic acid/g, described as ascorbic acid equivalent (EEA/g).

#### 2.5.2. Hydroxyl Radical Scavenging Activity Assay

The scavenging activity of plant extracts against the hydroxyl radical was measured based on Fenton's reaction (Fe^2+^ + H_2_O_2_ → Fe^3+^ + OH^−^ + OH^∙^). The results were expressed as an inhibition rate. Hydroxyl radicals were generated using a modified method [24], where 3 mL of 150 mM sodium phosphate buffer (pH 7.4) contained 10 mM FeSO_4_ × 7H_2_O, 10 mM EDTA, 2 mM sodium salicylate, 30% H_2_O_2_ (200 mL), and the plant extracts at 250 *μ*g/mL concentration. The positive control, the sodium phosphate buffer, was replaced by H_2_O_2_. The solutions were incubated at 37°C for 1 h, and the presence of the hydroxyl radical was detected by monitoring absorbance at 510 nm.

#### 2.5.3. Ferric Chelating

The ferrous ion chelating ability of plant extracts was measured according to [25]. The plant extract samples (250 *μ*g/mL concentration) were added to the mixture of 50 *μ*L of FeCl_2_ (2 mM) and 200 *μ*L of ferrozine (5 mM) in a final volume of 1 mL. This mixture was mixed well and then incubated for 10 min at room temperature. The absorbance was measured at 562 nm against a blank.

#### 2.5.4. Superoxide Radical Scavenging Activity Assay

The assay was based on the capacity of the leaf extracts to inhibit the photochemical reduction of nitroblue tetrazolium (NBT) in the riboflavin-light-NBT system [26]. Then, 3 mL reaction mixture (50 mM phosphate buffer (pH 7.8), 13 mM methionine, 2 mM riboflavin, 100 mM EDTA, and 75 mM NBT) was transferred to a tube, and then 1 mL from plant extract solution was added. The production of blue formazan was monitored as absorbance increased at 560 nm after a 10 min illumination from a fluorescent lamp. The entire reaction assembly was enclosed in a box covered by aluminum foil.

#### 2.5.5. Reducing Power

The reducing power of the samples was quantified as described previously [20, 27]. The plant extract at 250 *μ*g/mL concentration was added to the reaction mixture (0.2 M phosphate buffer (pH 6.6) and potassium ferricyanide (1% w/v)), with a final volume of 4 mL. This mixture was incubated at 50°C for 20 min. The reaction was terminated by adding the TCA solution (10% w/v). The solution was then mixed with distilled water and ferric chloride (0.1% w/v), and the absorbance was measured at 700 nm. The result was expressed as a percentage of the activity shown by 0.2 mg/mL of vitamin C.

#### 2.5.6. DPPH Free-Radical Scavenging Activity

The scavenging activity of the DPPH radical was assayed according to the method of Shimada et al. [28], with some modification. In a test tube, 1.5 mL of methanol was added, then the plant extract at 250 *μ*g/mL concentration, and finally 1 mL of a methanol solution of 0.1 mM DPPH (Fluka). Then, the mixture was shaken vigorously and allowed to stand at room temperature for 30 min. The absorbance was then measured at 517 nm. Lower absorbance of the reaction mixture indicates higher free-radical scavenging activity. The DPPH free-radical scavenging activity was calculated as follows: scavenging activity (%) = (1 − *A*_1_/*A*_0_) × 100, where *A*_0_ is the absorbance from the control and *A*_1_ is the absorbance from samples.

### 2.6. Cell Proliferation Analysis

HeLa, A549, 3T3, and CHO cells were grown in 75 cm^2^ culture flasks in Dulbecco's Modified Eagle Medium (DMEM). For the assay, cells were transferred into 96-well plates at a density of 5 × 10^3^ cell/well and were allowed to attach overnight in 200 *μ*L medium, at 37°C and 5% CO_2_. After this period, plant extracts were added at a final concentration of 100, 250, and 500 *μ*g/mL and for a period of 24 h, 48 h, or 72 h at 37°C and 5% CO_2_. After incubation, the plant extracts were removed by washing the cells twice with 200 *μ*L PBS, and 100 *μ*L of fresh medium was added. Then, 100 *μ*L of 5 mg/mL MTT (3-(4,5-dimethylthiazol-2-yl)-2,5-diphenyltetrazolium bromide) dissolved in DMEM was added to determine the effects of the samples on cell proliferation. The cells were then incubated for 4 h at 37°C and 5% CO_2_. To solubilize the product of MTT reduced, 100 *μ*L of ethanol was added to each well and thoroughly mixed using a multichannel pipette. Within 20 min of ethanol addition, the absorbance at 570 nm was read using a Multiskan Ascent Microplate Reader (Thermo Labsystems, Franklin, MA, USA). The percentage of cell proliferation was calculated as follows:

(1) $$\begin{array}{c} \%\;\text{cell proliferation}=\frac{\text{Abs. 570 nm of sample}}{\text{Abs. 570 nm of control}}\times 100. \end{array}$$

### 2.7. DAPI

Cells were seeded at a density of 2 × 10^6^ cells per well in 6-well plates. After 24 h of incubation, cells were cultured with leaf extract at 250 *μ*g/mL for 24 h at 37°C. Treated cells were washed with cold PBS following fixation in 4% paraformaldehyde at room temperature for 30 min. Then the cells were washed twice with PBS and maintained in PBS solution containing 0.1% Triton X-100 at room temperature for another 30 min. Samples were subsequently incubated in DAPI (1 *μ*g/mL) solution at room temperature for 30 min, washed with PBS, and examined under a fluorescence microscope within 24 h.

### 2.8. Annexin V-FITC Apoptotic Activity

To detect early apoptotic activity, an Annexin V-FITC apoptosis detection kit (Invitrogen, Catalog no. V13242) was used according to the manufacturer's instructions, with slight modifications. The cells were seeded at 8 × 10^4^ cells per well in 6-well plates. After treatment with 250 *μ*g/mL of leaf extract for 24 h, cells were harvested and washed twice with ice-cold phosphate-buffered saline (PBS) and resuspended in 100 *μ*L of binding buffer. A total of 5 *μ*L of Annexin V-FITC and 5 *μ*L of propidium iodide (PI) were added, and the mixture was incubated for 30 min in the dark. Finally, 400 *μ*L of binding buffer was added to the cells and the mixture was analyzed by flow cytometer (Becton Dickinson Co., San Jose, CA USA), using an FITC signal detector (FL1), and by PI staining with a phycoerythrin emission signal detector (FL2). The apoptotic percentage of 10,000 cells was determined, after which all the experiments reported in this study were performed 3 times. The data were analyzed using FlowJo software [29] for calculation of percentage cells with apoptosis per group.

### 2.9. Statistical Analysis

All data were expressed as means ± standard of triplicates. Each experiment was performed at least 3 times. Statistical analysis was performed by one-way ANOVA using the Graphpad Prism [30]. Tukey posttest was performed to compare different groups. Differences were considered significant when *P* value was less than 0.05.

## 3. Results and Discussion

### 3.1. Chemical Analysis

In the literature, only one report was found about the phytochemical screening of *P. volubilis*, performed by chemical reactions to identify the secondary metabolite present in hydroethanolic leaf extract [31]. Then, in this work, 5 leaf extracts obtained from *P. volubilis* were analyzed by TLC, which is an important tool, not only for the quality control of medicinal plants, but also for the analysis of herbal drugs [32]. Some bands with characteristic color of phenolic compounds, steroids, and/or terpenoids were observed in all extracts after the use of eluent systems and chromogenic agents described in item 2.3. Then, it were observed bands with color characteristics of steroids and/or terpenes in all extracts, yellow bands in MEL and EEL, and color bands characteristic of sugar in MEL, EEL, and AEL. When the plates were sprayed with natural reagent A, it was possible to observe the presence of yellow bands in MEL, EEL, and AEL, suggesting the presence of flavonoids. In addition, 3 flavonoids were used as standard. The color developed and the retention factor suggested the presence of kaempferol in MEL. After the use of ferric chloride, blue bands were observed especially in MEL, EEL, and AEL. Finally, after using the Dragendorff reagent, the development of orange bands, characteristic of the presence of alkaloids, was not observed in the plates. However, the absence of alkaloids needs to be confirmed because the five extracts analyzed in this work were not prepared by classic acid-basic extraction method to obtain alkaloids. The MEL and EEL were the extracts that showed a richer chemical profile. Our results are different from those published by Saavedra et al. [31] in that they described the presence of tannins and alkaloids in the *P. volubilis* leaf extract. This difference may be explained by different soil and environmental conditions during the plant growth, because their sample was collected in the city of Trujillo, Peru, as well as by the techniques used in their phytochemical analysis [33]. They used the classic chemical reactions, while our study analyzed the chemical composition of the extracts using TLC. 

Another analysis identified the chemical composition as shown in Table 1. A high concentration of sugar was observed in all five plant extracts. The total polyphenol content of the extracts was different depending on the type of extract. The highest concentration was found for CEL (10.8%) and then HEL (9.46%), followed by AEL (8.02%). The EEL and MEL had the lowest concentration compared to the other three, at 5.34% and 6.09%, respectively (Table 1). Some studies have shown that the antioxidant, anti-inflammatory, and antiproliferative activities may be related to the presence of flavonoids, terpenes, or phenolic compounds. In *Euphorbia fischeriana,* it was shown that antiproliferative activities were associated with terpenoids [34]. Duarte et al. [35] tested different phenolic compounds that were isolated from *Euphorbia lagascae, Euphorbia tuckeyana,* and *Pycnanthus* for antiproliferative activity and multidrug-resistant human cancer cells (MDR). Newman and Cragg [2] showed the importance of isolating and characterizing the activity from plant compounds, as at least 60% of chemical therapeutic agents come from plants.

### 3.2. Antioxidant Assay

 Antioxidants are compounds that can prevent biological and chemical substances from radical-induced oxidation damage. Because radical oxidation of substrates occurs through a chain reaction involving three stages (i.e., initiation, propagation, and termination), antioxidants show their effects through various mechanisms [36]. Thus, we used different methods to evaluate the effect of extracts on initiation (total antioxidant capacity, DPPH and reducing power), propagation (iron chelating), and termination (superoxide and hydroxyl radical scavenging activities) stages. 

 Thus, in order to evaluate the antioxidant activity, the five extracts from *P. volubilis* were analyzed by *in vitro* assays: total antioxidant capacity (TAC), hydroxyl radical scavenging activity, ferric chelating, superoxide radical scavenging, and reducing power. These different antioxidant assays were chosen, as the bioactive molecules present in these extracts may have different antioxidant activities, according to the assay. Three different concentrations of plant extract (100, 250, and 500 *μ*g/mL) were analyzed; however, the best results for these assays were obtained with the concentration of 250 *μ*g/mL.

**Figure 1:**
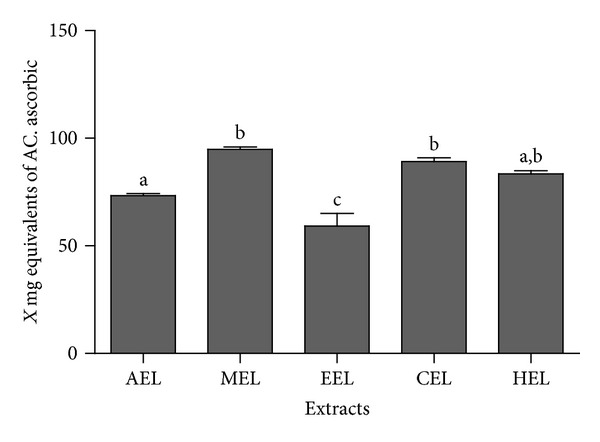
Total capacity antioxidant activity from plant extracts. The *y*-axis corresponds to mg of ascorbate acid equivalents (EEA/g) and the *x*-axis, the plant extract. EML corresponds to methanol extract, EHL to to hexane extract, ECL to chloroform extract, EEL to ethanol extract, and EAL to aqueous extract. The plant extract was used at 250 *μ*g/mL concentration. ^a,b,c^The different letters correspond to the significant data. The results were analyzed using ANOVA-Tukey test (*P* ≤ 0.05).

**Figure 2:**
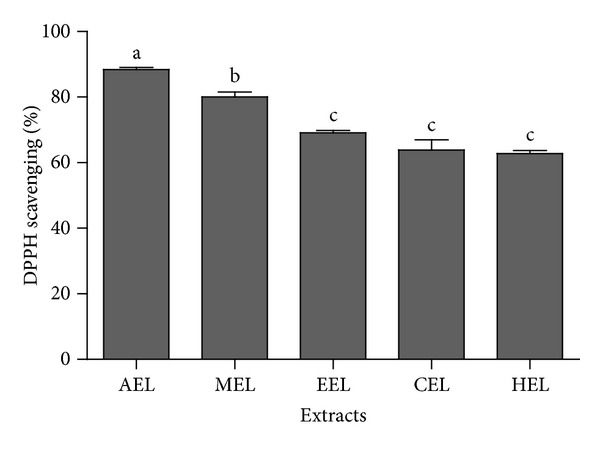
DPPH assay. The plant extract used for this assay was at 250 *μ*g/mL concentration. The graph to average ± standard deviation from DPPH scavenging. EML corresponds to methanol extract, EHL corresponds to hexane extract, ECL to chloroform extract, EEL to ethanol extract, and EAL to aqueous extract. The plant extract was used at 250 *μ*g/mL concentration. ^a,b,c^The different letters correspond to the significant data. The results were analyzed using ANOVA-Tukey test (*P* ≤ 0.05).

 The ferric chelating, superoxide radical scavenging, and reducing power assays did not show any significant activity. However, the TAC assay showed interesting results. It was verified with this assay that all five plant extracts had antioxidant activity (TAC), with values ranging from 59.31 to 97.76 EAA/g. Moreover, the better results for antioxidant activity were observed for MEL, HEL, and CEL: 97.76, 83.42, and 89.21 EAA/g, respectively (Figure 1). The other antioxidant assay evaluated was the DPPH.  Figure 2 shows the results obtained for the *P. volubilis* extracts at 250 *μ*g/mL. The values ranged from 62.8% to 88.3%. This assay measures the ability of the plant extract to donate an electron or H^+^ ion, and the results obtained were higher than 50% of what can be considered excellent, as the plant extract concentration was 250 *μ*g/mL. Guo et al. [37] worked with an ethanol extracts from *Tuber indicum* in order to have 80% activity in the DPPH assay. They used a concentration of 5 mg/mL from the ethanol extract; this concentration was 25-fold that of the *P. volubilis* extract concentration that was shown here. Furthermore, Basma et al. [38], working with extracts from *E. hirta* leaves, observed a DPPH scavenging activity of 72.9% using 1 mg/mL of extract. These DPPH values were similar to ours, but the extract concentration used was 4 times higher. Also, the methanol and ethanol extract from *Plukenetia conophora* showed 50% activity in the DPPH assay using an extract concentration of 62 *μ*g/mL and 69 *μ*g/mL [39], indicating that the genus *Plukenetia* may have antioxidant potential. 

 Peinado et al. [40], working with grape extract, proposed that sugars may also have a role as antioxidant molecules. Then, in order to analyze whether the sugar presented in the plant extracts may have any effect on the antioxidant activity, the Pearson correlation was done. a value was obtained for *R* of 0.72 when total phenolic was compared to TAC, reinforcing that the total phenolic content was responsible for the results obtained with TAC. When analyzed by DPPH assay, the value obtained was 0.6 for total phenolic and 0.57 for sugar, suggesting that for DPPH it was possible to have an additive effect for total phenolic and sugar.

### 3.3. Antiproliferative Activity

**Figure 3:**
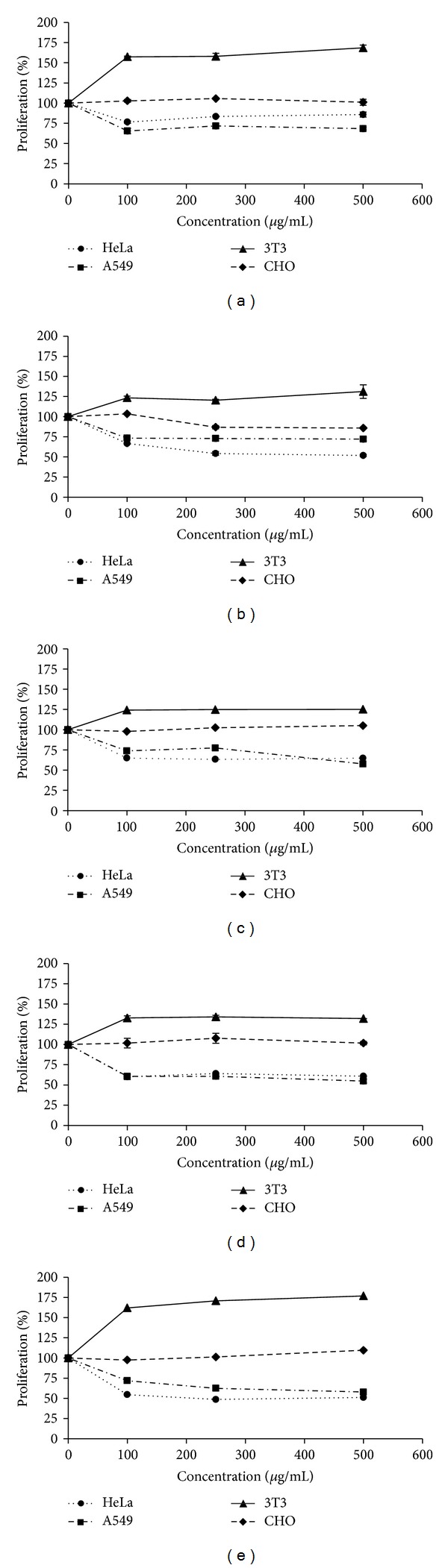
Antiproliferative activity from *Plukenetia volubilis* extracts. The *x*-axis corresponds to different plant concentrations used and the *y*-axis corresponds to the cell proliferation percentage. In (a) the aqueous extract is represented (AEL), (b) corresponds to MEL extract, (c) corresponds to EEL extract, (d) corresponds to CEL extract, and (e) corresponds to HEL extract. The data correspond to 48 h of cell exposure to the plant extract.

 It has been observed that extracts from the Euphorbiaceae family plant have potential antiproliferative activity for different tumor cell lines. Furthermore, the presence of phenolic compounds or terpenoids may be related to this antiproliferative activity [35, 41, 42]. Based on the antioxidant results, the proliferative or antiproliferative effects of *P. volubilis* extracts on different cell lines (normal cells: 3T3 and CHO; tumor cells: HeLa and A549) were analyzed. 3T3 cell line is a fibroblast cell from the Swiss mouse, CHO is a cell line derived from the Chinese hamster ovary, HeLa is a cervical cancer cell line, and A549 is a tumor cell line from lung tissue. For this assay, 3 extract concentrations (100, 250, and 500 *μ*g/mL) were tested at 3 different times (24 h, 48 h, and 72 h).  Figure 3 presents the graphs obtained in 48 h of the treatment with 3 extract concentrations (100, 250, and 500 *μ*g/mL). In this figure, some dose dependence was observed in the concentration of 100 *μ*g/mL and 250 *μ*g/mL. However, in the 500 *μ*g/mL concentration little difference was observed. Considering this observation, the data obtained only for the concentration of 250 *μ*g/mL at 3 different times: 24 h, 48 h, and 72 h was shown in Table 2. For the cell lineage A549, an inhibitory effect on the cell proliferation was observed for 24 h and 48 h. A similar effect was also observed for HeLa cell line for the MEL and HEL extracts, where the proliferation was observed at around 54–77% (Table 2). However, this inhibitory effect was not observed for the treatment of 72 h raising the hypothesis that perhaps in 72 h the bioactive molecules present in the extract were not active anymore. He et al. [43] observed that fractions obtained from *Panax notoginseng* were able to inhibit cervical cancer effectively. These authors found an IC_50_ of 166.8 and 276.9 *μ*g/mL for Caco-2 and LoVo cell lines, respectively [43]. The work presented here showed that for HeLa cells, the better extracts were MEL and HEL with a proliferation only of 54.3% and 48.5% (Table 2). Furthermore, for the A549 cell line, the better extracts were AEL, CEL, and HEL with a proliferation of 61.4%, 60.6%, and 61.5%, respectively, with a reduction of almost 40% (Table 2). Not all the extracts had the same inhibition pattern (Table 2). These data showed that the extracts from *P. volubilis *were able to reduce the proliferation for the two tumor cell lines tested by 40–50% rate. On the other hand, their effects on normal cells (3T3 and CHO) showed that these extracts had no cytotoxicity for these cells (Table 2). Furthermore, they showed an increase in proliferation on 3T3 cells (fibroblast cells). A significant increase was observed in the proliferation especially at 24 or 48 h (approximately 30–40% proliferation rate) (Table 2). However, this effect was not observed for CHO cells (Figure 3 and Table 2). For CHO, these extracts did not modify the proliferation rate, even when the extract concentration or time was changed. 

### 3.4. Nuclear Morphology Changes in HeLa Cells

**Figure 4:**
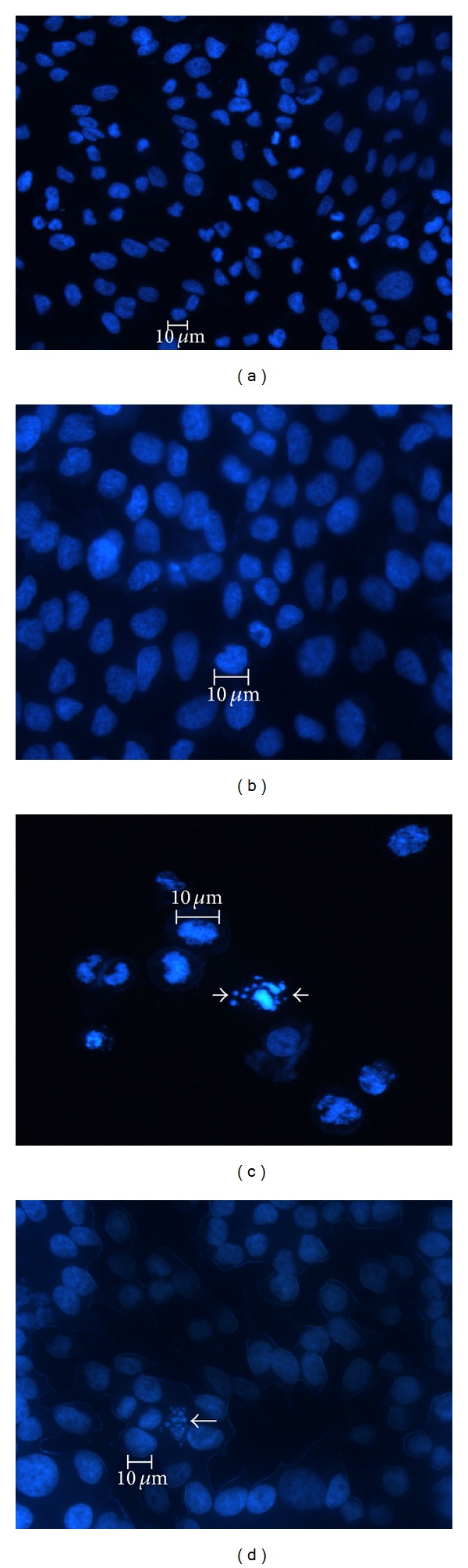
HeLa cell morphology after treatment with *P. volubilis *Extracts. HeLa cells were incubated with plant extracts at 250 *μ*g/mL concentration for 48 h and then were marked with DAPI to analyze the cell morphology. Arrows show regions where the cell had modification in its morphology. (a) corresponds to the control HeLa cells without plant extract, size 20x; (b) corresponds to HeLa cells without plant extract, size 40x; (c) corresponds to HeLa cells treated with EML extract, size 20x; (d) corresponds to HeLa cells treated with EHL, size 40x.

To evaluate the inhibition of the proliferation of the HeLa, a DAPI assay was performed. HeLa cells were previously treated with MEL and HEL extracts for 48 h, as the higher reduction was observed. The control used in this assay was the HeLa cells without plant extract. No change was observed in HeLa cell morphology; these cells were round and stained homogeneously with DAPI (Figures 4(a) and 4(b)). However, when the cells were treated with the *P. volubilis* extracts, changes were observed in the cell morphology such as pyknotic bodies, membrane blebbing, and nuclear condensation (Figures 4(c) and 4(d)). This morphology suggested that the inhibition in proliferation previously observed may be due to an apoptotic mechanism. Machana et al. [44] working with extracts from *Cladogynos orientales* (Euphorbiaceae) that had phenolic compounds and flavonoids, they observed a reduction on the proliferation rate of HepG2 tumor cells by early and late stages of apoptosis, similar to what was observed in this work. 

### 3.5. *P. volubilis *Extracts Induce Apoptosis or Necrosis

**Figure 5:**
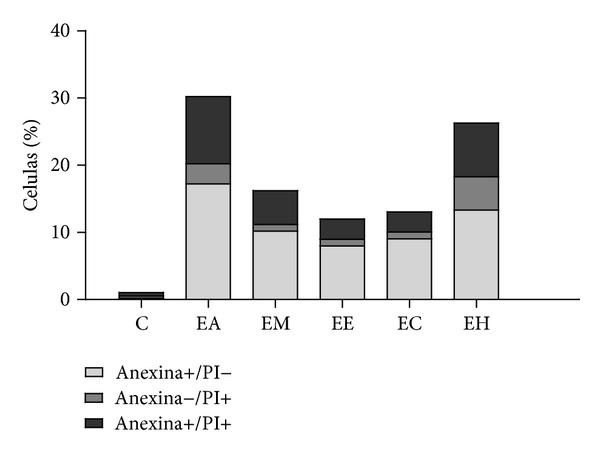
Distribution of apoptotic cells. HeLa cells were treated with 250 *μ*g/mL of plant extracts (AEL, MEL, EEL, CEL, or HEL) for 24 h and then stained with Annexine V-FITC and PI. C corresponds to HeLa cells not treated with plant extract.

The nuclear morphology changes previously observed may be a result of an apoptosis or necrosis process. In order to evaluate this, the HeLa cells were treated with *P. volubilis* extracts and then observed by flow cytometry analysis. This technique allowed observation of the cell phase and the presence of DNA fragments that were characteristic from the apoptosis process [45].  Figure 5 shows the results obtained when three groups of cells were observed. The first group was marked by Annexin V and not by PI. This group characterized the early apoptosis, where HEL, AEL, and MEL extracts had 13.3%, 17.2%, and 10.2% cells in early apoptosis respectively. The second group of cells was marked by Annexin V and PI, suggesting the late stages of the apoptosis process, where AEL and HEL extracts had 10.1% and 8.9%, respectively (Figure 5). And the third group of cells was marked only by PI, which corresponds to the necrosis process. This group was observed for AEL (3%) and HEL (5%) extracts (Figure 5). Studies done with *Croton lechleri *(Euphorbiaceae) showed the induction of the initial apoptosis in HeLa cells [46]. Mota et al. [47], using the ethanol extract from *Synadenium umbellatum* (Euphorbiaceae) on EAT cells, also found a proportion of the cells in apoptosis (29%) and also in necrosis (19.33%). Furthermore, the euphol (a triterpene) isolated from *Euphorbia tirucalli *(Euphorbiaceae) showed induction of apoptosis around 25% for gastric cancer cell lines CS12, AGS, and MKN45 [48, 49]. So, the results from *P. volubilis* extracts showed the potential of these biomolecules to induce apoptosis (early and late stages). 

## 4. Conclusions

Normally, in the cell, there is a balance between the free radicals produced by the organism and antioxidant products to keep this balance. ROS are also an important signal for the organism, but it is important to keep this balance. In disease conditions, so many free radicals may be generated that the antioxidant products cannot reduce the effect of such radicals. Thus, it is important to search for new bioactive molecules that can be used as a natural antioxidant and as the treatment for cancer and other diseases. The data presented here show that *Plukenetia volubilis *extracts had the presence of terpenoids, saponins, and phenolic compounds (flavonoids) that were known to have antioxidant, antiproliferative, and other activities. The data from CAT and DPPH indicated the potential of the AEL, MEL, and HEL extracts. Besides this, assays also verified an inhibitory effect on proliferation of HeLa and A549 cells, consequently producing early apoptosis and late apoptosis. However, these extracts were also able to induce proliferation of fibroblast cells, 3T3 cells. More studies are needed to understand the apoptosis pathway as well as the extract effects on fibroblast proliferation. 

## Figures and Tables

**Table 1:** Chemical composition.

Plant extract	Total Phenolic (%)	Sugar (%)	Protein (%)	Total (%)
AEL	8.02	91.49	0.49	100
MEL	6.08	92.88	1.04	100
EEL	5.34	93.44	1.23	100
CEL	10.85	84.17	4.98	100
HEL	9.46	86.94	3.60	100

**Table 2:** Proliferative effects from *Plukenetia volubilis* leaf extracts at 250 *µ*g/mL concentration on different cell lines in 24 h, 48 h and 72 h of incubation.

	HeLa	A549	3T3	CHO
AEL				
24 h	75.1 ± 2.3	61.4 ± 3.9	135.0 ± 4.3	102.9 ± 5.2
48 h	83.3 ± 0.5	71.6 ± 0.1	157.8 ± 3.7	105.6 ± 0.1
72 h	76.6 ± 1.8	90.6 ± 0.4	131.3 ± 2.1	103.8 ± 3.2
MEL				
24 h	66.6 ± 4.1	78.8 ± 2.6	130.9 ± 2.8	111.8 ± 5.6
48 h	54.3 ± 2.3	72.8 ± 2.9	120.4 ± 1.2	86.8 ± 2.1
72 h	78.0 ± 4.7	94.1 ± 3.6	117.1 ± 3.3	101.8 ± 5.6
EEL				
24 h	63.0 ± 1.6	76.8 ± 0.3	139.7 ± 0.7	99.0 ± 5.7
48 h	63.3 ± 1.7	77.4 ± 1.5	124.7 ± 0.3	102.4 ± 12.1
72 h	66.9 ± 3.0	103.3 ± 3.0	145.5 ± 0.6	104.2 ± 2.2
CEL				
24 h	58.0 ± 1.8	68.3 ± 3.6	131.9 ± 3.6	107.5 ± 6.1
48 h	64.0 ± 0.6	60.6 ± 0.9	134.0 ± 1.8	107.6 ± 5.1
72 h	59.3 ± 2.8	92.9 ± 2.6	142.5 ± 3.9	100.0 ± 5.0
HEL				
24 h	60.6 ± 2.1	61.5 ± 2.1	109.2 ± 1.0	100.2 ± 7.4
48 h	48.5 ± 1.1	62.4 ± 1.6	170.7 ± 1.8	101.2 ± 2.1
72 h	64.4 ± 5.2	84.6 ± 6.0	130.0 ± 2.3	99.6 ± 4.5
